# Implications of Activating the ANT2/mTOR/PGC-1α Feedback Loop: Insights into Mitochondria-Mediated Injury in Hypoxic Myocardial Cells

**DOI:** 10.3390/cimb45110543

**Published:** 2023-10-27

**Authors:** Meng Zhang, Yuanzhan Yang, Zhu Zhu, Zixuan Chen, Dongyang Huang

**Affiliations:** 1 Key Laboratory of Molecular Biology in High Cancer Incidence Coastal Chaoshan Area of Guangdong Higher Education Institutes, Department of Cell Biology and Genetics, Shantou University Medical College, Shantou 515041, China; mengzhangmd@126.com; 2 Beijing Key Laboratory for Separation and Analysis in Biomedicine and Pharmaceuticals, School of Medical Technology, Beijing Institute of Technology, Beijing 100081, China; yyzbit@163.com (Y.Y.); zx-chen@bit.edu.cn (Z.C.); 3 NHC Key Laboratory of Medical Immunology, Peking University, Beijing 100191, China; zhuzhu@bjmu.edu.cn; 4 Key Laboratory of Molecular Immunology, Chinese Academy of Medical Sciences, Beijing 100191, China

**Keywords:** acute myocardial infarction, oxygen–glucose deprivation, adenine nucleotide translocase 2, RNA interference, microRNA

## Abstract

Mitochondrial dysfunction is known to play a critical role in the development of cardiomyocyte death during acute myocardial infarction (AMI). However, the exact mechanisms underlying this dysfunction are still under investigation. Adenine nucleotide translocase 2 (ANT2) is a key functional protein in mitochondria. We aimed at exploring the potential benefits of ANT2 inhibition against AMI. We utilized an oxygen–glucose deprivation (OGD) cell model and an AMI mice model to detect cardiomyocyte injury. We observed elevated levels of reactive oxygen species (ROS), disrupted mitochondrial membrane potential (MMP), and increased apoptosis due to the overexpression of ANT2. Additionally, we discovered that ANT2 is involved in myocardial apoptosis by activating the mTOR (mechanistic target of rapamycin kinase)-dependent PGC-1α (PPARG coactivator 1 alpha) pathway, establishing a novel feedback loop during AMI. In our experiments with AC16 cells under OGD conditions, we observed protective effects when transfected with ANT2 siRNA and miR-1203. Importantly, the overexpression of ANT2 counteracted the protective effect resulting from miR-1203 upregulation in OGD-induced AC16 cells. All these results supported that the inhibition of ANT2 could alleviate myocardial cell injury under OGD conditions. Based on these findings, we propose that RNA interference (RNAi) technology, specifically miRNA and siRNA, holds therapeutic potential by activating the ANT2/mTOR/PGC-1α feedback loop. This activation could help mitigate mitochondria-mediated injury in the context of AMI. These insights may contribute to the development of future clinical strategies for AMI.

## 1. Introduction

Acute myocardial infarction (AMI) is an acute and severe ischemic heart disease that has emerged globally as a leading cause of mortality and morbidity worldwide [1]. Despite advancements in therapeutic strategies, patients often experience persistent retrosternal pain [2], arrhythmia [3], acute heart failure [4], cardiogenic shock [5], and other adverse cardiovascular events following AMI [6]. During AMI, the cardiomyocytes in the hypoxic–ischemic region undergo complex pathological changes and progressive cell death [7]. The prognosis of AMI is directly influenced by the extent of cardiomyocyte loss in the infarcted and border zone of the myocardium [8]. To enhance the effectiveness of AMI treatment, it is imperative to thoroughly investigate novel therapeutic targets at the cellular and molecular levels [9]. Addressing these unresolved challenges and concerns is crucial for improving outcomes in AMI patients.

Cardiomyocytes harbor a substantial number of mitochondria, which play a critical role in cell survival and death following AMI. During ischemic conditions, cardiomyocytes experience an accumulation of excessive reactive oxygen species (ROS) and a disruption in mitochondrial membrane potential (MMP), contributing to mitochondrial dysfunction [10]. The excess ROS is not conducive to maintaining mitochondrial integrity, affecting cell survival and protein maintenance [11]. Alterations in MMP further lead to the release of cytochrome C and activation of caspase. This eventually leads to the occurrence of apoptosis. Therefore, it becomes a viable therapeutic strategy that prevents mitochondria dysfunction under the hypoxic–ischemic condition [12]. Adenine nucleotide translocase (ANT) is the most abundant protein of the inner mitochondrial membrane; it facilitates the exchange of ADP and ATP across the mitochondrial inner membrane and plays an essential role in cellular energy metabolism [13,14,15]. It is reported that ANT is capable of stabilizing mitochondrial functions, such as sustaining the mitochondrial transmembrane potential [16]. There are multiple ANT isoforms with slightly different properties [17]. Adenine nucleotide translocase-1 (ANT1) is expressed in the heart–muscle–brain and has been shown to play a role in a variety of myocardial diseases. Heger et al. demonstrated that transgenic overexpression of the ANT1 protects cardiomyocytes against TGFβ_1_ -induced apoptosis by stabilization of the mitochondrial permeability transition pore (mPTP) [14]. Adenine nucleotide translocase-2 (ANT2) is expressed in most tissues and is inducible; it plays an important role in sustaining the mitochondrial transmembrane potential and inhibiting mitochondrial apoptosis [16]. Compared to ANT1, ANT2 has been proved to be more sensitive to hypoxia [18]. It is reported that the absence of ANT2 can induce the reduction in basal and maximal respiration [19]. In recent years, Jason et al. found that inactivation of ANT2 led to cardiac developmental failure because of its control capacity of the mPTP [17]. Gouriou et al. demonstrated that the absence of MFN2 can lead to the uncoupling of endoplasmic reticulum–mitochondria, resulting in limited calcium ion overload in mitochondria and triggering significant metabolic adaptation. This adaptation involves the upregulation of ANT2 to facilitate glycolytic ATP import and the upregulation of IF1 to restrict ATP degradation by F1FoATPase. Consequently, maintaining mitochondrial ATP levels and inner mitochondrial membrane polarization is crucial in preventing cell death caused by sustained hypoxia–reoxygenation insult [20]. However, the regulatory mechanisms of ANT2 in the cardiomyocyte injury induced by ischemia and hypoxia remain unclear during the progression of AMI.

RNA interfering (RNAi) is considered a fundamental mechanism that controls the flow of genetic information within cells including microRNA (miRNA) and small interfering RNA (siRNA). Both miRNA mimics and siRNAs share common characteristics, such as being double-stranded RNA molecules approximately 21 nucleotides long, binding to the Argonaute protein (AGO) to form an RNA-induced silencing complex (RISC), and inducing gene silencing in target genes [21]. Regarding therapeutic development, siRNA drugs have made significant advancements. Some siRNA-based drugs have gained FDA approval or are currently in late-stage clinical trials. On the other hand, the development of miRNA mimic therapeutics has been slower in comparison [22]. This field faces several challenges, including the identification of specific miRNAs with therapeutic potential, efficient delivery of miRNA mimics to target tissues, and the possibility of off-target effects, which makes miRNAs promising targets for the development of new drugs and the diagnosis of a variety of AMI [23,24].

In this study, we investigated the mechanisms involved in AMI and identified the highly conserved ANT2 as an antiapoptotic factor in the infarct zones of mice hearts following AMI, as well as in AC16 cells subjected to OGD. ANT2 was found to directly induce multiple components of a common pathway associated with mitochondria-mediated apoptosis. In addition, we demonstrated the efficacy of RNAi technology, including siRNA and miRNA, in human AC16 cardiocytes to determine the optimal therapeutic approach for the treatment of AMI. Our findings shed light on a novel mechanism underlying AMI and offer promising prospects for future clinical strategies in its treatment.

## 2. Materials and Methods

### 2.1. Data Integration for Differentially Expressed miRNAs (DEMs)

To identify potential miRNAs for the diagnosis and treatment of AMI, we conducted a search using the keyword “acute myocardial infarction” on the GEO (https://www.ncbi.nlm.nih.gov/geo/ (accessed on 7 April 2020)) and Arrayexpress (https://www.ebi.ac.uk/biostudies/arrayexpress (accessed on 3 February 2022)) databases. We screened two datasets, namely, GSE148153 and E-MEXP-3578, with the species type set as “Homo sapiens” and the study type selected as “noncoding RNA profiling by high-throughput sequencing” or “RNA-seq of noncoding RNA”, respectively. In GSE148153, we selected seven samples (GSM4455137, GSM4455138, GSM4455139, GSM4455140, GSM4455141, GSM4455142, and GSM4455143) as the control group and five samples (GSM4455160, GSM4455161, GSM4455162, GSM4455163, and GSM4455164) as the AMI group. In dataset E-MEXP-3578, we chose nine healthy subjects as the control group and thirteen AMI patients as the AMI group. This resulted in a total of 18 serum samples in the AMI group and 16 in the control group. To identify key miRNAs involved in AMI, we integrated the differentially expressed miRNAs (DEMs) from both datasets using the R package “Linear Models for Microarray Data (limma)”. The selection criteria for the key miRNAs included a log|FoldChange| > 1 for both datasets and an adjusted *p*-value (adjust *p*) < 0.05 as threshold values.

### 2.2. Cells Culture

Cardiomyocyte AC16 cells were obtained from the BeNa Culture Collection (BNCC, Henan, China) and cultured in high-glucose DMEM (Gibco, Waltham, MA, USA) supplemented with 10% FBS (Gibco), 1% penicillin–streptomycin solution (Bio-channel, Nanjing, Jiangsu, China) in a humidified incubator in an atmosphere of 95% air and 5% CO_2_ at 37 °C. The cells were cultured in T25 flasks (NEST, Jiangsu, China) for 24 h for subsequent experiments.

### 2.3. Oxygen and Glucose Deprivation (OGD) Cells Models

To establish the OGD cell models, AC16 cells were first washed with PBS and then exposed to an OGD buffer with the following composition in millimolar (mM): 115 NaCl, 4 KCl, 1 KH_2_PO_4_, 1.25 CaCl_2_, 1 MgSO_4_, pH 6.8. The cells were then placed inside a hypoxia chamber (Billups-Rothenberg, San Diego, CA, USA). The chamber was flushed with a mixed gas consisting of 95% N2 and 5% CO_2_ for a duration of 30 min. Following this, the chamber was sealed and the cells were incubated for an additional 6, 12, 24, and 48 h. The control group, known as the normal group, consisted of AC16 cells that were maintained under normoxic conditions with standard medium.

### 2.4. Cell Transfection

ANT2 siRNA and miR-1203 mimics were synthesized and verified by Tsingke Biotechnology Co., Ltd., Beijing, China. For transfection, 50 nM of ANT2 siRNA were transfected with Omifection-R (Omiget, Beijing, China) for a duration of 48 h. Similarly, 50 nM of miR-1203 mimics were transfected with Omifection-R (Omiget, Beijing, China) for 48 h each. The ANT2 overexpression plasmid with pcDNA 3.1 vector was also synthesized and verified by Tsingke Biotechnology Co., Ltd., China. AC16 cells were cotransfected with control/ANT2 OE plasmid (500 ng/μL) and control mimic/miR-1203 mimics (50 nM) using Omifection/Omifection-R (Omiget, Beijing, China).

### 2.5. Luciferase Reporter Assays

The 3’UTR sequence of ANT2, which contains the potential binding sites for miR-1203, was cloned into the pmirGLO vector by Tsingke Biotechnology Co., Ltd. Specifically, the sequence CUCCGG in the 3’UTR was mutated to GAGGCC. AC16 cells were cotransfected with WT/MUT vectors (500 ng/μL) and control mimic/miR-1203 mimics (50 nM) using Omifection/Omifection-R (Omiget, Beijing, China). After 48 h of transfection, AC16 cells were collected to measure Firefly and Renilla luciferase activity using the Dual-Lumi™ II Luciferase Reporter Gene Assay Kit (Beyotime, Shanghai, China) following the provided protocols.

### 2.6. Measurement of Mitochondrial Membrane Potential (MMP)

The measurement of MMP was conducted using a mitochondrial membrane potential assay kit with JC-1 (Beyotime, Shanghai, China) following the provided protocol. AC16 cells were seeded on 12-well plates and, after 24 h, subjected to different durations of OGD. To measure MMP, the cells were washed with PBS and incubated in serum-free DMEM containing (1×) JC-1 staining working solution at 37 °C for 20 min. Subsequently, the cells were washed twice with (1×) JC-1 buffer, and 1 mL of DMEM was added. Finally, the cells were visualized and imaged using a fluorescence microscope (BX61; Olympus Corporation, Tokyo, Japan).

### 2.7. Intracellular ROS Levels

The levels of intracellular ROS were assessed using an ROS assay kit (Beyotime, Shanghai, China) following the provided protocols. Initially, AC16 cells were seeded on 12-well plates and allowed to adhere for 24 h. Subsequently, the cells were subjected to different durations of OGD treatment. Following the OGD treatment, AC16 cells were washed with PBS and incubated in serum-free DMEM containing 0.1% 2′,7′-dichlorofluorescein diacetate (DCFH-DA) at 37 °C for a duration of 20 min. Afterward, the cells were washed three times with serum-free DMEM and images were captured using a fluorescence microscope.

### 2.8. Flow Cytometry

The apoptosis rate was determined using the Annexin V-FITC/PI kit (Beyotime, Shanghai, China) and analyzed on a cytoFLEX LX flow cytometer (Beckman-Counter Electronics, Suzhou, Jiangsu, China) with the assistance of CytExpert V.2.4.0.28 software. AC16 cells were labeled with FITC-Annexin V and PI for 15 min in the dark. The fluorescence data obtained from the flow cytometer were utilized to establish quadrants on Annexin V/PI plots, enabling the identification of apoptotic cells using the formula (Annexin V+/PI−, Annexin V+/PI+). The apoptotic rate was calculated as the percentage of apoptotic cells out of the total cells, multiplied by 100%.

### 2.9. Animal AMI Model

To establish the AMI mice model, a total of 15 male mice with a wild-type background on a C57BL/6 strain were used. These mice were aged between 7 to 8 weeks. Among the 15 mice, 9 were selected and divided into two groups: a control group consisting of 7 mice and an AMI group consisting of 8 mice. To induce anesthesia, all mice were injected with a pentobarbital sodium solution (6 mg/mL) intraperitoneally at a dose of 0.1 mL/10 g of body weight. The mice were considered to be deeply anesthetized when they remained immobile for 2 min. Following anesthesia, a left thoracotomy was performed to induce left anterior descending (LAD) occlusion in the AMI group. This occlusion was maintained for a duration of 72 h. In contrast, the control group underwent the same surgical procedure without LAD occlusion. Electrocardiogram recordings were taken before and after the surgery. After the 72 h occlusion period, the mice were euthanized by inhalation of 25% CO_2_ until respiratory and cardiac arrest occurred. The hearts were then isolated and washed with PBS for imaging to visualize the infarct area. Tissue samples from the infarct area were isolated. It is important to note that all animal experiments conducted in this study were approved by the Ethical Committee of the Beijing Institute of Technology, Beijing, China (permit #: SYXK-BIT-20200107001), and were performed in accordance with the guidelines for the Care and Use of Laboratory Animals. The chapter 3 “Environment, Housing, and Management” provides guidelines for the environment, housing, and management of laboratory animals used or produced for research, testing, and teaching.

### 2.10. Hematoxylin–Eosin (HE) Staining

HE staining was conducted on both control and AMI mice to evaluate myocardial injury by examining morphological changes in myocardial tissue. The tissues were dehydrated using anhydrous ethanol, embedded in paraffin, sectioned, baked, deparaffinized, counterstained with HE, sealed with neutral glue, and observed under a microscope.

### 2.11. Electrocardiography

The baseline and end-point electrocardiographic (ECG) analysis was captured from standard lead II limb leads via a digital ECG machine (ECG-1103GVet, Shenzhen Carewell Electronics Co., Ltd., Shenzhen, China) during the surgical process for control and AMI mice. The presence of ST-segment elevation on the ECG indicated that myocardial infarction occurred. A trained technician evaluated and screened the ECG parameters for spontaneous cardiac arrhythmias in the AMI mouse.

### 2.12. Total RNA Extraction and Real-Time Quantitative PCR (RT-qPCR)

Total RNA from the cells was extracted by the TRIzol method (Invitrogen, Waltham, MA, USA). A total of 2 μg total RNA was used for reverse transcription using a Universal RT-PCR Kit (M-MLV) (Solarbio, Beijing, China). The quantitative PCR was performed with Talent qPCR PreMix (SYBR Green) (TIANGEN, Beijing, China) on a Gentier 96 real-time PCR system (Tianlong, Shanxi, China). U6 small nuclear RNA (U6) and GAPDH mRNA were used as internal controls for miRNA and mRNA. At least three independent runs were performed for each sample. The miRNA and mRNA expression levels were analyzed using the threshold cycle 2^−△△Ct^ method. All primers were designed manually and synthesized by Sangon Biotech. The primer sequences are listed in Table 1.

### 2.13. Western Blot

Proteins were isolated from cells and tissues using RIPA lysis buffer (Solarbio, Beijing, China) supplemented with phenylmethyl sulfonyl fluoride (PMSF; Solarbio, Beijing, China) and protein phosphatase inhibitor (Solarbio, Beijing, China), following the provided protocol. Equal amounts of protein (30 μg) from each sample were loaded for the Western blot assay. β-tubulin was used as an internal standard to normalize protein levels. The primary antibodies used for Western blotting were β-tubulin (1:1000, A12289, ABclonal), ANT2 (1:1000, A15639, ABclonal, Wuhan, China), Bax (1:1000, A20227, ABclonal, China), Bcl-2 (1:1000, A20736, ABclonal, China), mTOR (1:1000, A23168, ABclonal, China), PGC-1α (1:1000, A19674, ABclonal, China), and caspase-3 (1:1000, A19654, ABclonal, China). The Western blot bands were quantified using ImageJ 1.53a software, and the results were presented as fold change normalized to the control group.

### 2.14. Prediction of miRNAs Targeted ANT2

The prediction of miRNAs targeted ANT2 were analyzed through TargetScan 7.2 databases (https://www.targetscan.org/vert_72/ (accessed on 1 March 2018)).

### 2.15. Statistical Analysis

The statistical analysis of all data was performed using GraphPad Prism 9.3.1 software (GraphPad Prism, La Jolla, CA, USA). The results are presented as the mean ± standard deviation (S.D.). Normality and lognormality tests in GraphPad Prism were used to check whether all datasets fulfilled normal distribution for parametric tests. Results showed that all datasets fulfilled normal distribution for parametric tests. For comparisons between two groups, the two-tailed unpaired Student’s *t*-test method was utilized. In cases where multiple groups were involved, two-way analysis of variance (ANOVA) was employed for data comparison. A *p*-value of ≤0.05 was considered statistically significant.

## 3. Results

### 3.1. Myocardial Cells Injury Induced by OGD In Vitro and by Ischemia–Hypoxia In Vivo

**Figure 1:**
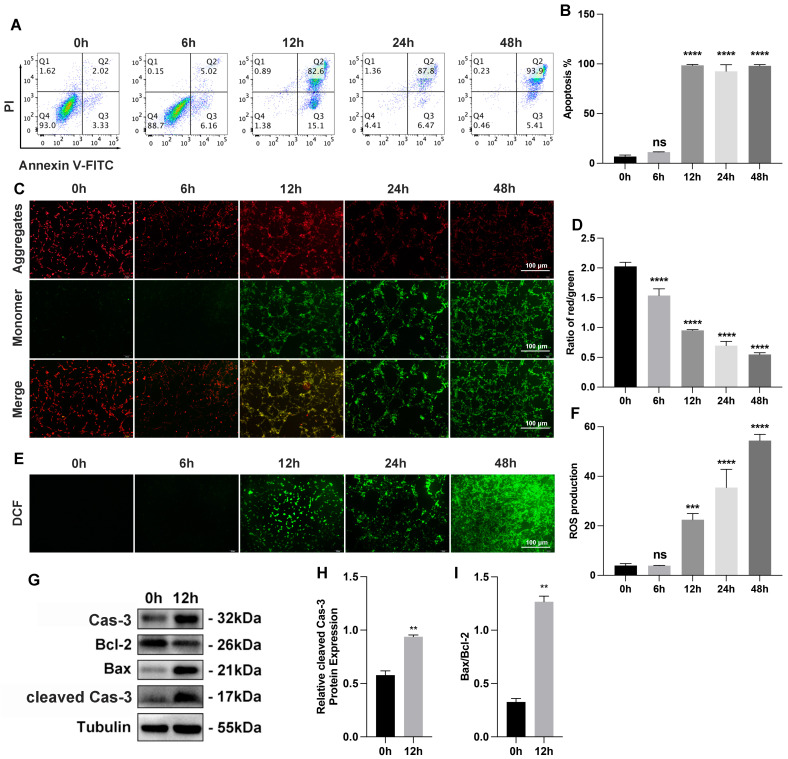
Myocardial cell injury induced by OGD (oxygen–glucose deprivation) in vitro. (**A**) Apoptosis of AC16 cells determined by FACS (fluorescence-activated cell sorting) analysis with annexin V and PI staining; n = 3 per group. (**B**) Quantification data of AC16 cell apoptosis; n = 3 per group. (**C**) Representative photography of MMP under different OGD times with JC-1 staining (aggregate red; monomer green). Scale bars, 100 μm; n = 3 per group. (**D**) Quantification data of MMP (mitochondrial membrane potential) measurement; n = 3 per group. (**E**) Representative photography of intracellular ROS levels under different OGD times with DCFH-DA staining (green). Scale bars, 100 μm; n = 3 per group. (**F**) Quantification data of intracellular ROS (Reactive oxygen species) levels measurement; n = 3 per group. (**G**–**I**) Bax, Bcl-2, caspase-3, and cleaved caspase-3 protein expression by Western blot analysis in 12 h OGD cells models; n = 3 per group. ** *p* < 0.01, *** *p* < 0.001, **** *p* < 0.0001; ns: no significance.

We investigated the myocardial cell injury following the establishment of OGD-treated AC16 cells and mice model of AMI. For the OGD-treated cell model, AC16 cells were subjected to different durations of OGD ranging from 0 h to 48 h. Flow cytometry analysis confirmed that the apoptosis rate in OGD-treated AC16 cells increased as the duration of OGD was extended (Figure 1A,B). In our study, we utilized the JC-1 molecule probe to assess the MMP in AC16 cells, which serves as an indicator of early apoptosis. The JC-1 probe has the ability to aggregate in the mitochondrial matrix and emit red fluorescence. When the MMP decreases, the JC-1 probe remains in a monomeric state, leading to green fluorescence. Following a 12 h treatment of AC16 cells with OGD, we observed an increase in green fluorescence, indicating a decrease in MMP and suggesting an elevation in AC16 cell apoptosis (Figure 1C,D). We also measured intracellular ROS levels of AC16 cells treated with different OGD times. We observed a significant increase in ROS levels in AC16 cells as the duration of OGD extended, particularly after 12 h, indicating the progression of severe OGD-induced damage (Figure 1E,F). Western blot analysis revealed apoptosis in the 12 h OGD cell models (Figure 1G–I).

**Figure 2:**
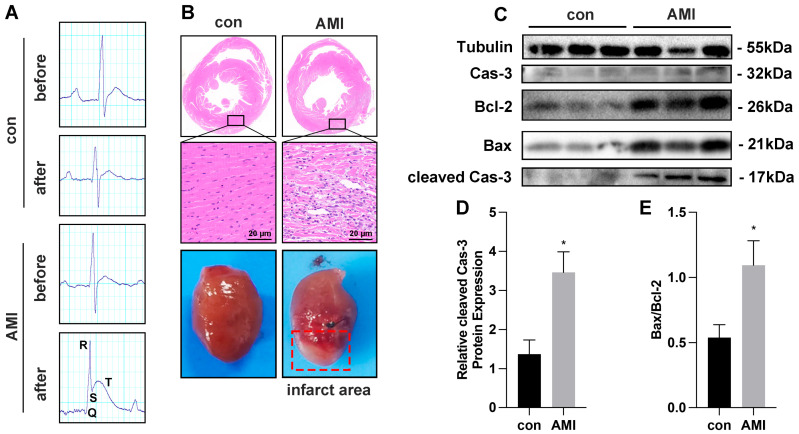
Myocardial cell injury induced by ischemia–hypoxia in vivo. (**A**) The electrocardiogram of control (n = 4) and AMI (Acute myocardial infarction) mice (n = 5) before and after the modeling process. (**B**) Representative histopathological images of HE staining of heart infarct area in control and AMI mice. Scale bars, 20 μm; n = 3 per group. (**C**–**E**) Bax, Bcl-2, caspase-3, and cleaved caspase-3 protein expression by Western blot analysis in infarct area cells from both control (n = 4) and AMI mice (n = 5). * *p* < 0.05.

In the case of AMI mice models, we successfully established the models as evidenced by ST-segment elevation in the electrocardiogram (ECG) results (Figure 2A). Histological damage to myocardial cells after 72 h of LAD occlusion was assessed using HE staining (Figure 2B). In the control group, the cardiomyocytes were arranged neatly, the cytoplasm was abundant and uniform, and the interstitium was normal. However, in the AMI group, some myocardial nuclei were lost, the cardiomyocytes were vacuolated, the myocardial tissue in the infarction area was disordered, and the cardiomyocytes disappeared in the infarction area, which were replaced by fibrous scar tissue. Infract area of isolated heart in AMI group could be observed obviously (Figure 2B). Western blot analysis was conducted to evaluate the expressions of Bax, Bcl-2, and cleaved caspase-3 in the myocardial tissue (Figure 2C–E). The expression of cleaved caspase-3 and the ratio of Bax/Bcl-2 in the AMI group exhibited a significant increase compared to the control group, indicating the occurrence of apoptosis in myocardial cells from the AMI mice.

### 3.2. ANT2 siRNA Shows a Protection for AC16 Cells from Injury Caused by OGD

**Figure 3:**
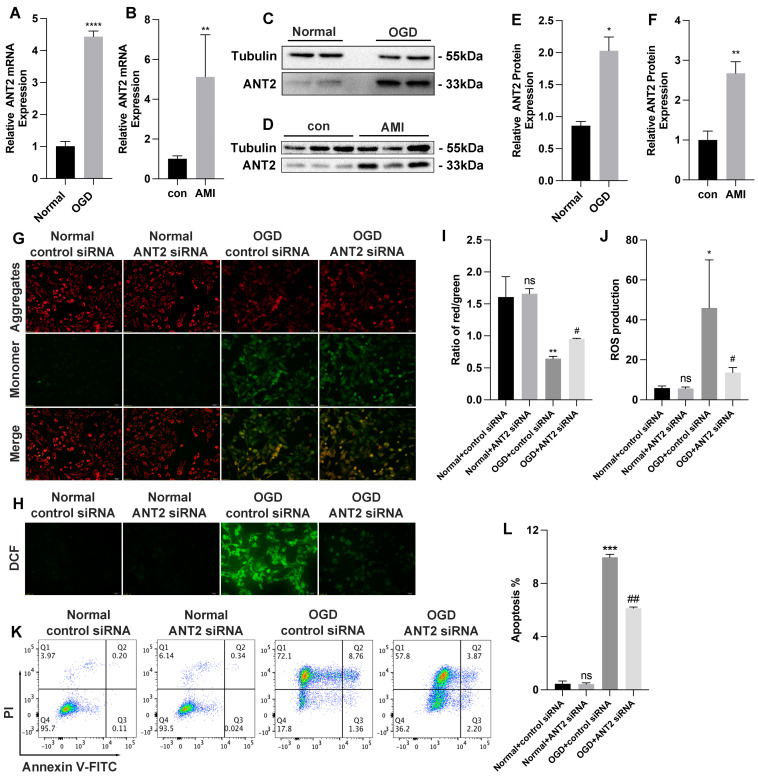
The inhibition of ANT2 (Adenine nucleotide translocase 2) protects AC16 cells from injury caused by OGD. (**A**) ANT2 mRNA quantitation by RT-qPCR in AC16 cells under 12 h OGD conditions; n = 3 per group. (**B**) ANT2 mRNA quantitation by RT-qPCR in AMI mice infarct area cells; n = 4 in control group, n = 5 in AMI group. (**C**) ANT2 protein expression by Western blot analysis in AC16 cells under 12 h OGD conditions; n = 3 per group. (**D**) ANT2 protein expression by Western blot analysis in AMI mice infarct area cells; n = 4 in control group, n = 5 in AMI group. (**E**) ANT2 protein expression by gray analysis of Western blot band in AC16 cells under 12 h OGD conditions; n = 3 per group. (**F**) ANT2 protein expression by gray analysis of Western blot band in AC16 cells under 12 h OGD conditions; n = 3 per group. (**G**) Representative photography of MMP in AC16 cells transfected with control siRNA or ANT2 siRNA in both normal and OGD groups by JC-1 staining (aggregate red; monomer green). Scale bars, 100 μm; n = 3 per group. (**H**) Representative photography of intracellular ROS levels in AC16 cells transfected with control siRNA or ANT2 siRNA in both normal and OGD groups by DCFH-DA staining (green). Scale bars, 100 μm; n = 3 per group. (**I**) Quantification data of MMP measurement; n = 3 per group. (**J**) Quantification data of intracellular ROS levels measurement; n = 3 per group. (**K**) Apoptosis of AC16 cells transfected with control siRNA or ANT2 siRNA in both normal and OGD groups was determined by FACS (fluorescence-activated cell sorting) analysis with annexin V and PI staining; n = 3 per group. (**L**) Quantification data of AC16 cell apoptosis; n = 3 per group. * *p* < 0.05, ** *p* < 0.01, *** *p* < 0.001, **** *p* < 0.0001, # *p* < 0.05, ## *p* < 0.01 (#: vs. OGD+ control siRNA); ns: no significance.

We firstly examined the expression of ANT2 at the mRNA level in cells subjected to 12 h of OGD conditions (Figure 3A) and in cells from the infarct area of AMI mice (Figure 3B). The results demonstrated a significant increase in ANT2 expression both in OGD cells and AMI mice. Western blot analysis validated these findings at the protein level (OGD cells: Figure 3C,E; AMI mice: Figure 3D,F). To investigate the role of ANT2 in apoptosis, we utilized siRNA to knock down ANT2 expression in AC16 cells. Following transfection with control siRNA and ANT2 siRNA for 48 h, the cells were exposed to OGD for 12 h. We assessed the impact of ANT2 on MMP and ROS levels. Our results using MMP-JC1 staining demonstrated that the decreased MMP levels in the OGD group were restored upon transfection with ANT2 siRNA, showing higher MMP levels compared to cells transfected with control siRNA in the OGD group (Figure 3G,I). These findings indicate that the inhibition of ANT2 played a crucial role in mitigating apoptosis damage in AC16 cells induced by OGD. Notably, the inhibition of ANT2 did not have any adverse effects on normal mitochondrial function, as evidenced by the absence of significant alterations in ROS levels within the normal group (Figure 3H,J). Furthermore, flow cytometry analysis demonstrated a decrease in the apoptosis rate of OGD-induced AC16 cells following ANT2 siRNA transfection compared to control siRNA transfection (Figure 3K,L). These findings suggest that inhibiting ANT2 could potentially offer protection to AC16 cells against OGD-induced damage. These results provide support for the essential role of ANT2 inhibition in safeguarding cardiac myocytes against OGD-induced injury.

**Figure 4:**
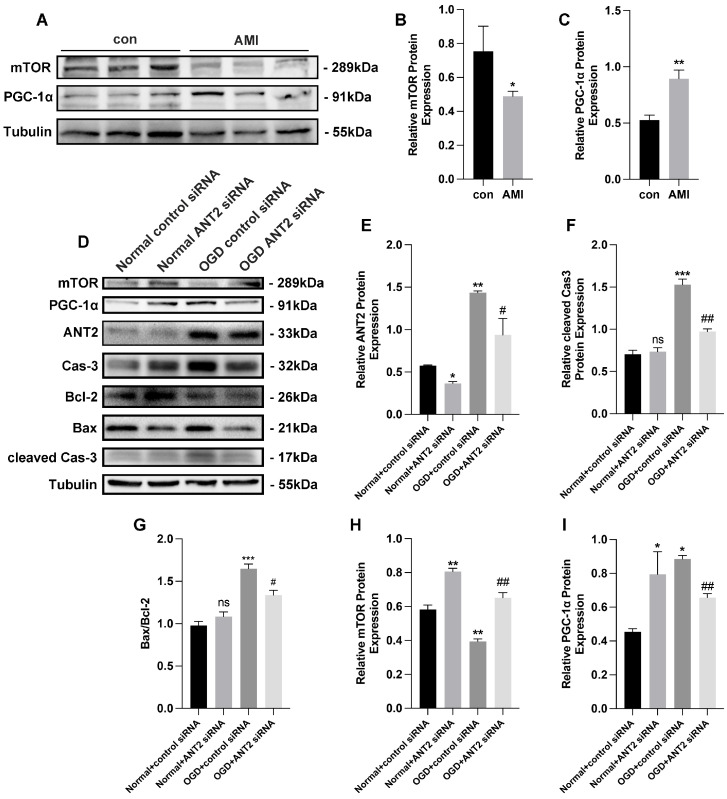
The inhibition of ANT2 inhibited the ANT2/mTOR/PGC-1α feedback loop. (**A**–**C**) mTOR (mechanistic target of rapamycin kinase) and PGC-1α (PARG coactivator 1 alpha) protein expression by Western blot and gray analysis in AMI mice infarct area cells; n = 4 in control group, n = 5 in AMI group. (**D**–**I**) mTOR, PGC-1α, ANT2, caspase-3, Bax, Bcl-2, and cleaved caspase-3 protein expression by Western blot in AC16 cells transfected with control siRNA or ANT2 siRNA in both normal and OGD groups; n = 3 per group. * *p* < 0.05, ** *p* < 0.01, *** *p* < 0.001, # *p* < 0.05, ## *p* < 0.01 (#: vs. OGD + control siRNA); ns: no significance.

In addition, we observed lower levels of mTOR and higher levels of PGC-1α in the infarct area of AMI mice compared to the control group (Figure 4A–C). This led us to hypothesize that the differential expression of ANT2 may influence the expression of mTOR and PGC-1α. To investigate this regulatory mechanism in an ANT2-dependent manner, we conducted further analysis using a Western blot in an in vitro model under OGD conditions (Figure 4D). Initially, we reduced the expression of ANT2 through the transfection of ANT2 siRNA, which significantly increased when AC16 cells were exposed to OGD conditions (Figure 4E). Additionally, we observed a significant increase in the expression of cleaved caspase-3 and the ratio of Bax/Bcl-2, both of which are associated with apoptosis, in AC16 cells after OGD treatment, indicating OGD-induced injury. However, in AC16 cells transfected with ANT2 siRNA under OGD conditions, the expression of cleaved caspase-3 and the ratio of Bax/Bcl-2 decreased, suggesting that the inhibition of ANT2 protected cardiac myocytes from OGD-induced injury (Figure 4F,G). Furthermore, we found that the expression of mTOR decreased and PGC-1α increased under OGD conditions (Figure 4H,I), providing further evidence of the interconnected regulatory relationship between ANT2, mTOR, and PGC-1α.

### 3.3. ANT2 Was Targeted and Regulated by miR-1203 in Human AC16 Cardiocytes

**Figure 5:**
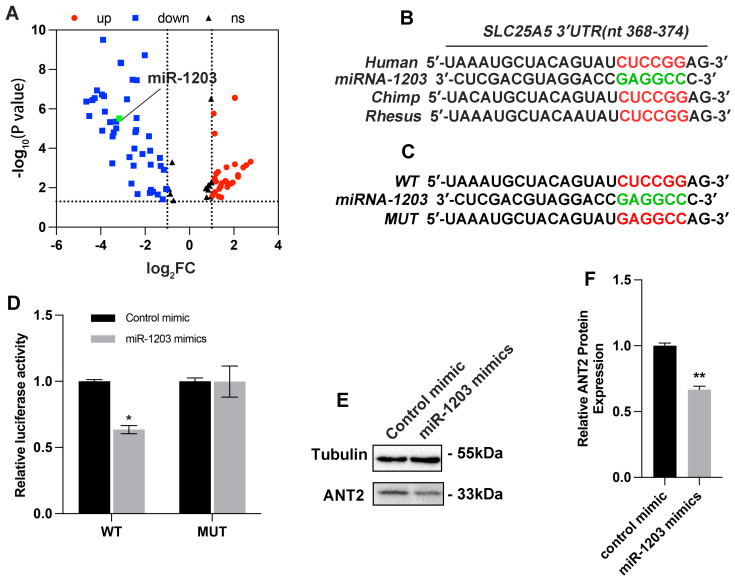
miR-1203 targeted and regulated the expression of ANT2. (**A**) Volcano map showing the 145 miRNAs commonly differentially expressed from the two datasets; the green dots represent miR-1203 (log2FC < −3, low expression). (**B**) Schematic representation of predicted miR-1203 binding sites on 3′UTR of ANT2 in humans. (**C**) Schematic representation of wild-type and mutated ANT2 3′UTR site. (**D**) AC16 cells transfected with wild-type or mutated ANT2 3′UTR luciferase constructs and control mimic or miR-1203 mimics; n = 3 per group. (**E**,**F**) ANT2 protein expression by Western blot analysis and gray analysis in AC16 cells transfection with miR-1203 mimics; n = 3 per group. * *p* < 0.05, ** *p* < 0.01, ns: no significance.

To screen out the key miRNAs with significant changes that might target ANT2, we conducted a search for miRNA sequencing data from the serum of patients experiencing AMI. Ultimately, two datasets (E-MEXP-3578 and GSE148153) were selected for analysis of significant miRNAs. Based on the findings presented in Figure 5A, we selected the top 20 miRNAs that exhibited significant changes for further analysis. Through our analysis, we identified that only miR-1203 targeted ANT2 and it binds to a specific region (position 368–374) within the 3’UTR of the *SLC25A5* gene encoding ANT2 (Figure 5B). To validate the targeting of miR-1203 with ANT2, additional luciferase reporter assays were performed. AC16 cells were cotransfected with wild-type (WT) or mutated (MUT) vectors, along with control mimic or miR-1203 mimics, and incubated for 48 h. The results revealed that miR-1203 mimics led to a reduction in luciferase activity specifically in the wild-type construct, while no significant effect was observed in the mutated construct (Figure 5C,D). Furthermore, the protein expression of ANT2 notably decreased following transfection with miR-1203 mimics (Figure 5E,F). These findings provide further evidence supporting the conclusion that the overexpression of miR-1203 can inhibit the expression of ANT2.

### 3.4. miR-1203 Mimics Show Potential as Protective Agents against OGD-Induced Injury in AC16 Cells

**Figure 6:**
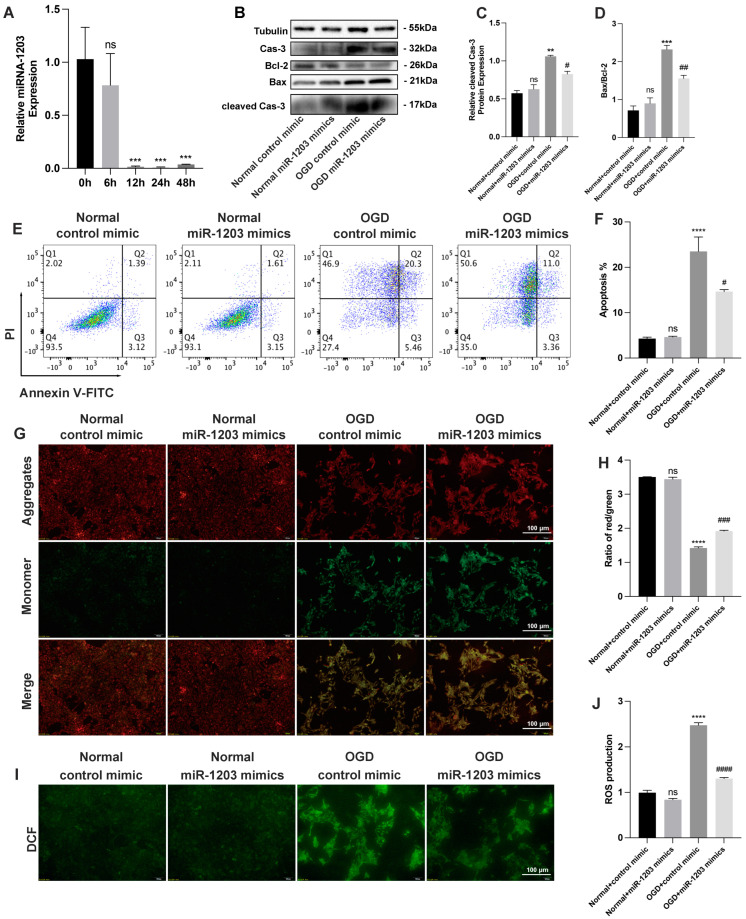
Overexpression of miR-1203 protected AC16 cells from OGD-induced injury. (**A**) miR-1203 quantitation by RT-qPCR in AC16 cells after OGD treatment for 0–48 h; n = 3 per group. (**B**–**D**) Bax, Bcl-2, caspase-3, and cleaved caspase-3 protein expression by Western blot analysis; n = 3 per group. (**E**) Apoptosis of AC16 cells transfected with control mimic or miR-1203 mimics in both normal and OGD groups determined by FACS (fluorescence-activated cell sorting) analysis with annexin V and PI staining; n = 3 per group. (**F**) Quantification data of AC16 cell apoptosis; n = 3 per group. (**G**) Representative photography of MMP in AC16 cells transfected with control mimic or miR-1203 mimics in both normal and OGD groups by JC-1 staining (aggregate red; monomer green). Scale bars, 100 μm; n = 3 per group. (**H**) Quantification data of MMP measurement; n = 3 per group. (**I**) Representative photography of intracellular ROS levels in AC16 cells transfected with control mimic or miR-1203 mimics in both normal and OGD groups by DCFH-DA staining (green). Scale bars, 100 μm; n = 3 per group. (**J**) Quantification data of intracellular ROS levels measurement; n = 3 per group. ** *p* < 0.01, *** *p* < 0.01, **** *p* < 0.0001, # *p* < 0.05, ## *p* < 0.01, ### *p* < 0.001, #### *p* < 0.0001 (#: vs. OGD + control mimic); ns: no significance.

In AC16 cells subjected to time-dependent OGD treatment, we observed a significant decrease in the expression of miR-1203 (Figure 6A). To assess the effects of miR-1203 in AC16 cells, we subjected the cells to OGD conditions for 12 h after transfecting them with either a control mimic or miR-1203 mimics for 48 h. Overexpression of miR-1203 in AC16 cells resulted in a downregulation of cleaved caspase-3 expression and a decrease in the Bax/Bcl-2 ratio under OGD conditions (Figure 6B–D). Furthermore, flow cytometry analysis demonstrated a reduction in the apoptosis rate of OGD-induced AC16 cells following miR-1203 transfection compared to control mimic transfection (Figure 6E,F). These findings suggest that the increased expression of miR-1203 can protect AC16 cells from OGD-induced damage. To further explore the protective effects of miR-1203 on AC16 cells during OGD conditions, we assessed the alterations in MMP and ROS. Our results using MMP-JC1 analysis revealed that the decreased levels of MMP and JC-1 molecular probes as monomers, as well as the increased levels of apoptosis in AC16 cells following OGD treatment, were restored upon transfection with miR-1203 mimics in the OGD groups. These cells exhibited higher MMP compared to cells transfected with control mimic in the OGD groups (Figure 6G,H). Based on our findings, it is suggested that the overexpression of miR-1203 played a significant role in reducing apoptosis damage in AC16 cells treated with OGD. In the normal group, there was no significant difference in MMP and JC-1 molecular probe aggregation in the mitochondrial matrix after transfection with either control mimic or miR-1203 mimics. Similarly, our ROS results also supported these findings. In the normal groups, whether transfected with control mimic or miR-1203 mimics, there was no significant difference in intracellular ROS levels. However, after 12 h of OGD treatment, the intracellular ROS levels in AC16 cells significantly increased, indicating OGD-induced injury. Interestingly, following transfection with miR-1203 mimics, there was a significant decrease in ROS levels (Figure 6I,J), supporting the protective effect of miR-1203 overexpression on AC16 cells against OGD-induced injury.

### 3.5. ANT2 Overexpression Counteracted the Effect of miR-1203

**Figure 7:**
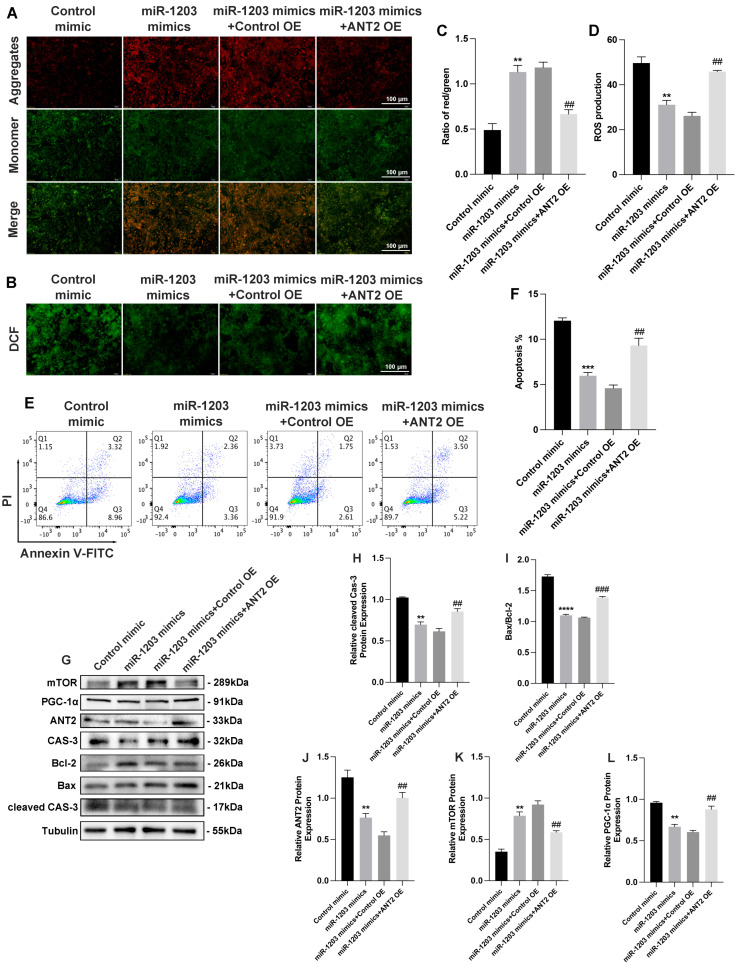
ANT2 overexpression counteracted the effect of miR-1203. (**A**) Representative photography of MMP in AC16 cells transfected with ANT2 plasmid and/or miR-1203 mimics by JC-1 staining (aggregate red; monomer green). Scale bars, 100 μm; n = 3 per group. (**B**) Representative photography of intracellular ROS levels in AC16 cells transfected with ANT2 plasmid and/or miR-1203 mimics by DCFH-DA staining (green). Scale bars, 100 μm; n = 3 per group. (**C**) Quantification data of MMP measurement; n = 3 per group. (**D**) Quantification data of intracellular ROS levels measurement; n = 3 per group. (**E**) Apoptosis of AC16 cells transfected with ANT2 plasmid and/or miR-1203 mimics determined by FACS (fluorescence-activated cell sorting) analysis with annexin V and PI staining; n = 3 per group. (**F**) Quantification data of AC16 cell apoptosis; n = 3 per group. (**G**–**L**) Western blot analysis of signal pathway proteins mediated by ANT2. ** *p* < 0.01, *** *p* < 0.001, **** *p* < 0.0001, ## *p* < 0.01, ### *p* < 0.001 (##: vs. miR-1203 mimics + control OE); ns: no significance.

**Figure 8:**
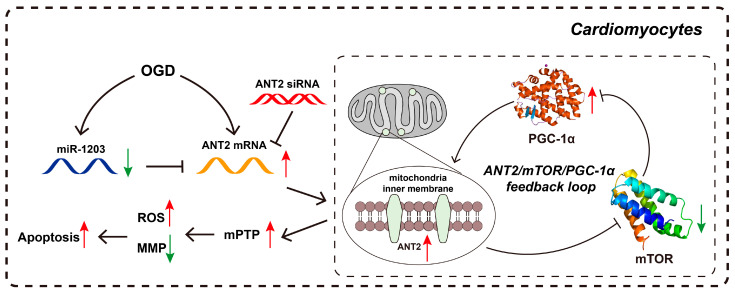
Schematic representation of the ANT2/mTOR/PGC-1α feedback loop in cardiomyocytes during AIM. ANT2 expression increases in cardiomyocytes during hypoxia. The overexpression of ANT2 leads to the opening of the mPTP, which in turn produces higher levels of ROS and disrupts MMP, leading to increased apoptosis. Additionally, the ANT2/mTOR/PGC-1α feedback loop further enhances the expression of ANT2. Overexpression of miR-1203 or knockdown ANT2 through siRNA protects cardiomyocytes from OGD-induced injury by targeting ANT2 and reducing its expression. (Red arrow: increased level; green arrow: decreased level).

Furthermore, to explore the functional interplay between miR-1203 and ANT2, we examined the impact of miR-1203 on AC16 cell apoptosis in the context of ANT2 overexpression. Based on our observations, we found that the protective effect of miR-1203 led to the restoration of MMP and ROS levels. However, this restoration was counteracted by ANT2 overexpression (Figure 7A–D). Flow cytometry assay results further demonstrated that the suppressed apoptosis of AC16 cells under OGD conditions, induced by miR-1203 overexpression, was partially counteracted by ANT2 overexpression (Figure 7E,F). Furthermore, ANT2 overexpression counteracted the effect of miR-1203, resulting in a reversal of the ratio of Bax/Bcl-2 and the expression levels of cleaved caspase-3, mTOR, and PGC-1α (Figure 7G–L). Based on these findings, we concluded that miR-1203 overexpression can confer protective effects on AC16 cells under OGD conditions by regulating ANT2, thereby offering a potential target for the treatment of AMI. Based on these findings, we proposed the existence of a regulatory mechanism involving the miR-1203 or ANT2 siRNA, which may modulate the ANT2/mTOR/PGC-1α feedback loop (Figure 8).

## 4. Discussion

The increased risk of AMI in the general population is a global concern, attributed to various genetic and nongenetic factors such as poor lifestyle, hypertension, diabetes, and obesity. These factors contribute to cardiomyocyte injury caused by acute or persistent ischemia and hypoxia [25,26]. Despite extensive research on protective targets for the heart, there is a pressing need for more effective strategies to reduce the area of AMI. Cardiomyocytes, being rich in mitochondria, play a crucial role in meeting the high-energy metabolism demands of the heart [12]. However, under hypoxic conditions, the function of mitochondria is disrupted, leading to myocardial necrosis and other cardiac abnormalities [27]. Our study supported these findings by demonstrating that OGD enhances apoptosis in AC16 cells, accompanied by decreased MMP and increased intracellular levels of ROS in a time-dependent manner. We hypothesized that under hypoxic conditions, the opening of the mPTP allowed the influx of calcium ions (Ca^2+^) and other ions into the mitochondrial matrix, leading to MMP loss, elevated ROS levels, mitochondrial dysfunction, and cellular apoptosis [28]. Furthermore, our results indicated increased apoptosis in myocardial cells in both in vivo and in vitro models of AMI due to oxygen deprivation, highlighting the importance of mitochondrial recovery in mitigating the negative effects of AMI.

ANT2 has been shown to regulate mitochondrial membrane potential [13] and transport ATP/ADP [29]. A study by Peng et al. showed that ANT2 plays a significant role in cellular apoptosis, and their ANT2 downregulation has resulted in protective effects against apoptosis in brown adipocytes through increasing NF-kB activity [30]. Our findings also supported the protective role of the decreased ANT2 in AC16 cells against OGD by inhibiting the apoptosis of cardiomyocytes. Furthermore, we found that the inhibition of ANT2 can upregulate mTOR levels, subsequently blocking the expression of PGC-1α, a critical factor for mitochondrial metabolism and biogenesis. Recently, Hang et al. also reported that PGC-1α can be inhibited by the activation of mTORC1, deteriorating the mitochondrial functions [31]. It is important to note that PGC-1α, which is activated by mTOR, can also enhance the transcription of ANT2 [32]. Based on the information provided, it is reasonable to hypothesize that the ANT2/mTOR/PGC-1α pathway may function as a feedback loop that could potentially alleviate myocardial injury during AMI. Further research and investigation would be needed to validate and explore this hypothesis in order to gain a better understanding of its potential implications in the context of AMI.

RNAi is a prominent gene regulation mechanism in biology, involving the suppression of gene expression by RNA. This process encompasses miRNA and siRNA, which effectively reduce gene expression through various mechanisms, including mRNA degradation, inhibition of mRNA translation, and chromatin remodeling [33]. The potential of miRNA as an RNAi technique for effective treatments in various cardiac disorders, including AMI, has garnered significant attention among researchers, as evidenced by multiple reports [34,35,36,37,38]. For instance, Ma et al. reported that overexpressed miR-150 had attenuated hypoxia-induced apoptosis of cardiomyocytes by downregulating the RP94 gene [39]. Similarly, miR-27a-5p exhibited cardioprotective effects by preventing H9c2 cells from hypoxia-induced damage by targeting the Atg7 gene [40]. In the present study, we found that the lowered expression of miR-1203 was detected in AC16 cells following OGD, which eventually reversed hypoxia-induced cardiomyocyte damage when expressed at higher levels. The same expression pattern of miR-1203 has also been reported in the serum of children with combined pituitary hormone deficiency (CPHD) and some prostate cancer patients [41,42]. Moreover, Xu et al. indicated that miR-1203 could prevent the human endometrial cells following oxygen and glucose deprivation reoxygenation (OGDR) treatment-induced necrosis by targeting cyclophilin D and inhibiting mitochondrial depolarization [43]. In recent years, RNAi based therapeutics have become one of the most promising directions to repair damaged myocardium in myocardial disease. Tan et al. reported an miR-21-based therapy to reprogram macrophages post myocardial ischemia–reperfusion injury to improve myocardial remodeling [44]. Our results showed that ANT2 has the potential to be a therapeutic target for AMI. RNAi therapy targeting ANT2 might become one of the methods for clinical treatment of AMI in the future.

Given the complex nature of cellular physiological and pathological regulatory processes, it is plausible to consider that the activation of ANT2 may also be involved in other signaling pathways. It is important to acknowledge that our findings represent only one of the significant pathways associated with ANT2. In future research, we intend to explore alternative environments, such as the microgravity environment in space, to further investigate the mechanism of ANT2 action. This approach holds potential for uncovering the intricate mechanisms mediated by ANT2. The utilization of the microgravity environment in space has emerged as a valuable method for studying novel biomolecular mechanisms across various fields of research [45,46]. Additionally, by leveraging artificial intelligence-based multiomics analysis, our objective is to investigate the intricate pathological mechanisms of AMI mediated by ANT2. This research endeavor aims to provide insights into the role of ANT2 in the accelerated progression of the disease during AMI [47,48,49,50] and explore potential treatment approaches based on the RNAi pathway.

## 5. Conclusions

In conclusion, our study demonstrates that the upregulation of ANT2 disrupts the regulation of mitochondrial membrane potential, leading to elevated levels of ROS and ultimately initiating apoptosis in myocardial cells through the ANT2/mTOR/PGC-1α feedback loop mechanism during AMI. Moreover, the inhibition of ANT2, particularly through RNAi therapy using hsa-miR-1203 or siRNA, contributes to a decrease in myocardial apoptosis during AMI. These findings highlight the importance of investigating ANT2 as a potential target for improving treatment approaches for AMI.

## Figures and Tables

**Table 1:** Primer sequences of gene and miRNA for RT-qPCR.

Gene/miRNA	Primer Sequences (5′-3′)
U6	Forward: GCTTGCTTCGGCAGCACATATAC
	Reverse: TGCATGTCATCCTTGCTCAGGG
hsa-miR-1203	Forward: GGAGCCAGGATGCAGCTC
	Reverse: GCGACACAGAATTATACGACTCAC
GAPDH	hsa-Forward: TCATTGACCTCAACTACATGGT
	hsa-Reverse: CTAAGCAGTTGGTGGTGCAG
	mmu-Forward: TCATTGACCTCAACTACATGGT
	mmu-Reverse: CTAAGCAGTTGGTGGTGCAG
ANT2	hsa-Forward: CTACTTTGCAGGGAATCTGG
	hsa-Reverse: GACACGTTAAAGCCTTGGTAC
	mmu-Forward: CTACTTTGCAGGGAACCTGG
	mmu-Reverse: GACACATTAAAGCCTTGGTAC

## Data Availability

The datasets used and/or analyzed during the current study are available from the corresponding author upon reasonable request.
